# Dataset of ^18^O and ^2^H in streamflow across Canada: A national resource for tracing water sources, water balance and predictive modelling

**DOI:** 10.1016/j.dib.2021.106723

**Published:** 2021-01-09

**Authors:** J.J. Gibson, P. Eby, T.A. Stadnyk, T. Holmes, S.J. Birks, A. Pietroniro

**Affiliations:** a InnoTech Alberta, 3-4476 Markham Street, Victoria BC V8Z 7X8 Canada; b University of Victoria, Department of Geography, Victoria BC V8W 3R4 Canada; c University of Calgary, Geography, 2500 University Drive NW, Calgary AB T2N 1N4 Canada; d University of Manitoba, Civil Engineering, Winnipeg MB R3T 5V6 Canada; e InnoTech Alberta, 3608 - 33 St NW Calgary, Alberta T2L 2A6 Canada; f National Hydrological Service, Meteorological Service of Canada, National Hydrology Research Centre, 11 Innovation Blvd., Saskatoon SK S7N 3H5 Canada

**Keywords:** Stable isotopes, Oxygen-18, Deuterium, Isotope mass balance, Streamflow, Watershed, Canada

## Abstract

Oxygen-18 and deuterium were measured in streamflow samples collected from 331 gauging stations across Canada during 2013 to 2019. This dataset includes 9206 isotopic analyses made on 4603 individual water samples, and an additional 1259 analysis repeats for quality assurance/quality control. We also include arithmetic and flow-weighted averages, and other basic statistics for stations where adequate data were available. Station data are provided including station code, name, province, latitude, longitude and drainage area. Flow data were extracted from the historical database of the Water Survey of Canada. Details on the preliminary application of these data are provided in “^18^O and ^2^H in streamflow across Canada” [1]. Overall, these data are expected to be useful when combined with precipitation datasets and analytical or numerical models for water resource management and planning, including tracing streamflow source, water balance, evapotranspiration partitioning, residence time analysis, and early detection of climate and land use changes in Canada.

## Specifications Table

**Table:** 

Subject	Hydrology and Water quality
Specific subject area	Baseline streamflow stable isotope dataset for use in regional hydrology and watershed investigations across Canada
Type of data	Tables, figure, .xlsx file
How data were acquired	Water samples were collected by hydrometric staff of the Water Survey of Canada during regular visits to stream gauges across Canada. Samples for isotopic analysis were collected in tightly sealed 30 mL high-density polyethylene (HDPE) bottles and were stored at room temperature and shipped to avoid freezing prior to analysis. A dual-inlet Thermofisher Scientific Isotope Ratio Mass Spectrometer (IRMS), Delta V was used for oxygen-18 and deuterium analysis. Oxygen-18 was measured using a Gasbench peripheral whereas deuterium was measured using an H-Device.
Data format	Raw isotope analytical data are reported in per mil relative to Vienna Standard Mean Ocean Water (‰ VSMOW) and normalized to SMOW/SLAP (Standard Light Antarctic Precipitation).
Parameters for data collection	Water samples were collected from mid-channel locations at mid-depth within the river water column at each gauging station to ensure representativeness of discharge. If this was not possible due to ice conditions or other safety issues, staff typically collected samples from an adjacent bank, avoiding poorly mixed zones below tributaries and/or backwater areas. Alternately, water was collected during ice-on conditions from a borehole augered in the ice. Metadata such as station number, sampling date, time, backwater effects, and ice conditions were routinely recorded.
Description of data collection	Co-location of water isotope sampling with stream gauging by Water Survey of Canada (https://wateroffice.ec.gc.ca) was important to allow for flow-weighting of the isotope composition of discharge, which is a preferred method for obtaining long-term isotope values for continental runoff. The survey represents the first national survey of isotopes in streamflow for both ^18^O and ^2^H.
Data source location	Canada-wide, 331 stations within Water Survey of Canada network.
Data accessibility	With the article
Related research article	J.J., Gibson, T. Holmes, T. Stadnyk, S.J., Birks, P. Eby, A. Pietroniro, 2020. ^18^O and ^2^H in streamflow across Canada. Journal of Hydrology:Regional Studies 32, 100754. https://doi.org/10.1016/j.ejrh.2020.100754[1].

## Value of the Data

• This is the first Canada-wide baseline stable isotope dataset for rivers.
• This dataset enables identification of water sources and distributions in streamflow and can help to resolve spatial and/or temporal variations in potential water sources including precipitaiton (snow, rain, mixed), groundwater, lakes and wetlands [2], [3], [4], [5].
• This dataset facilitates the estimation of water balance of catchment areas [6], [7], [8] using the spatiotemporal distributions in precipitation [9,10] as well as evaporation-transpiration partitioning [11], [12], [13], catchment residence times [14], and assessment of water-carbon cycle linkages [11,15], and nutrients [16].
• Isotope data provide process evaluation targets and diagnostic tools for refining water-cycle modeling capabilities at the local, regional and continental scales [17], [18], [19], [20]. Improvements in model prediction can potentially advance representation of hydrological processes and pathways, forecasting of water availability including floods and droughts, and as an early warning of hydrologic changes occurring due to land use impacts and climate change.

## 1 Data Description

**Fig. 1:**
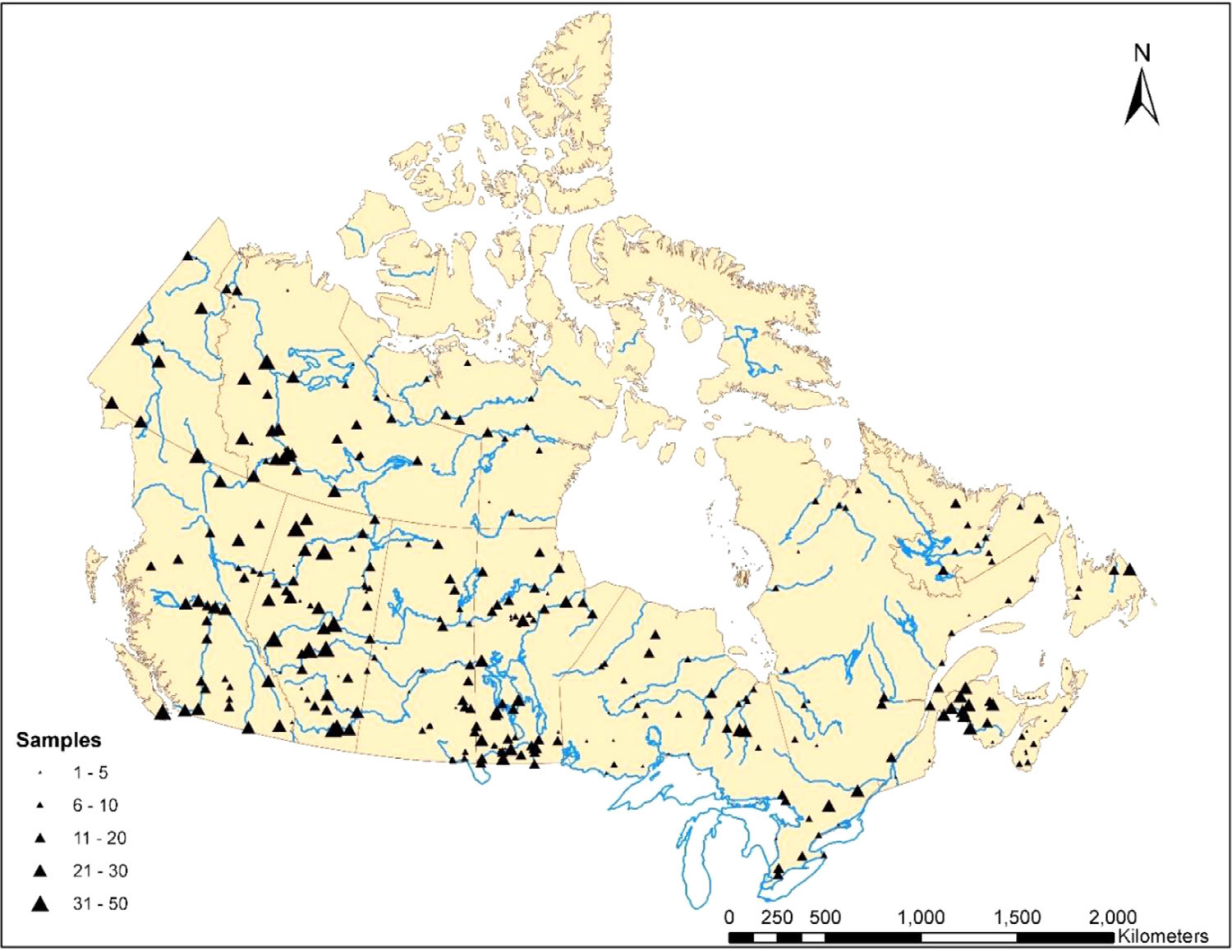
Map of Canada showing the Water Survey of Canada river isotope monitoring network, 2013-2019, where size of station symbols is proportionate to the number of samples analyzed (after Gibson et al. 2020 [1]).

Analytical isotope data for ^18^O and ^2^H are provided for individual samples collected during the survey at 331 stations (Fig. 1) operated by Water Survey of Canada during regular site visits by staff. Data include raw analytical results and routine repeats, mean sample results, station arithmetic means, analytical uncertainty, and lab and field comments. In addition, flow-weighted values for ^18^O and ^2^H for 161 stations, as well as drainage basin areas (km^2^), mean flow (m^3^·s^−1^), water yield (mm·yr^−1^) and station elevation (m.a.s.l.) are also provided (mmc1.xlsx). Summaries of isotope results by station (georeferenced) are provided for each province/territory (Table 1, Table 2, Table 3, Table 4, Table 5, Table 6, Table 7, Table 8, Table 9, Table 10, Table 11, Table 12, Table 13).

**Table 1:** Summary of isotope monitoring data for British Columbia.

					Mean		Max.		Min.		Flow weighted	
Station Name	Station ID	Latitude	Longitude	Samples	δ^18^O	δ^2^H	δ^18^O	δ^2^H	δ^18^O	δ^2^H	δ^18^O	δ^2^H
`FINLAY RIVER ABOVE AKIE RIVER'	`07EA005′	57.08	−125.15	15	−20.47	−159.23	−19.87	−154.81	−21.29	−166.35	n.d.	n.d.
`PEACE RIVER AT HUDSON HOPE'	`07EF001′	56.03	−121.91	9	−19.11	−148.76	−18.58	−146.39	−19.70	−151.77	n.d.	n.d.
`PEACE RIVER ABOVE PINE RIVER'	`07FA004′	56.20	−120.81	1	−19.64	−150.89	−19.64	−150.89	−19.64	−150.89	n.d.	n.d.
`PINE RIVER AT EAST PINE'	`07FB001′	55.72	−121.21	18	−18.78	−144.57	−18.12	−138.59	−19.75	−151.54	−19.00	−146.05
`MURRAY RIVER NEAR THE MOUTH'	`07FB002′	55.55	−121.20	3	−19.26	−148.11	−18.60	−143.43	−19.82	−150.59	n.d.	n.d.
`PEACE RIVER NEAR TAYLOR'	`07FD002′	56.14	−120.67	9	−19.20	−148.51	−18.75	−144.37	−19.65	−152.91	n.d.	n.d.
`PEACE RIVER ABOVE ALCES RIVER'	`07FD010′	56.13	−120.06	9	−19.23	−149.93	−18.52	−145.21	−20.85	−161.90	n.d.	n.d.
`BABINE RIVER AT OUTLET OF NILKITKWA LAKE'	`08EC013′	55.43	−126.70	15	−16.25	−132.53	−15.66	−128.64	−17.37	−138.06	−16.50	−133.95
`SKEENA RIVER AT USK'	`08EF001′	54.63	−128.43	14	−17.56	−133.70	−16.32	−125.47	−18.81	−142.92	−17.97	−136.55
`COWICHAN RIVER NEAR DUNCAN'	`08HA011′	48.73	−123.71	39	−10.39	−74.46	−9.68	−69.98	−11.04	−78.57	−10.50	−74.84
`NECHAKO RIVER BELOW CHESLATTA FALLS'	`08JA017′	53.69	−124.84	25	−15.61	−126.13	−15.35	−124.61	−15.99	−128.20	−15.65	−126.19
`NECHAKO RIVER AT VANDERHOOF'	`08JC001′	54.03	−124.01	29	−15.60	−127.51	−15.17	−124.92	−16.22	−131.90	−15.59	−127.73
`NECHAKO RIVER AT ISLE PIERRE'	`08JC002′	53.96	−123.23	17	−16.19	−132.59	−15.91	−130.36	−16.69	−136.62	−16.21	−132.84
`FRASER RIVER AT HANSARD'	`08KA004′	54.08	−121.85	21	−18.59	−142.79	−17.31	−135.37	−21.79	−167.71	−19.12	−145.34
`FRASER RIVER AT SHELLEY'	`08KB001′	54.00	−122.62	24	−18.69	−143.08	−17.10	−130.97	−20.38	−155.68	−19.23	−146.50
`FRASER RIVER AT SOUTH FORT GEORGE(Frozen 1984)'	`08KE018′	53.90	−122.73	18	−16.69	−135.06	−15.98	−130.56	−18.08	−142.87	n.d.	n.d.
`WEST ROAD RIVER NEAR CINEMA'	`08KG001′	53.31	−122.89	18	−17.20	−140.13	−16.52	−137.35	−18.45	−147.47	−17.43	−141.46
`NORTH THOMPSON RIVER AT MCLURE'	`08LB064′	51.04	−120.24	5	−18.10	−138.56	−17.83	−136.87	−18.43	−140.69	n.d.	n.d.
`SOUTH THOMPSON RIVER AT CHASE'	`08LE031′	50.76	−119.74	8	−17.01	−130.86	−16.39	−128.37	−17.42	−132.39	n.d.	n.d.
`THOMPSON RIVER NEAR SPENCES BRIDGE'	`08LF051′	50.35	−121.39	15	−17.65	−134.85	−17.11	−130.37	−18.41	−139.17	−17.80	−135.88
`FRASER RIVER NEAR MARGUERITE'	`08MC018′	52.53	−122.44	15	−18.06	−140.36	−17.21	−133.22	−19.05	−145.33	−18.45	−142.15
`FRASER RIVER AT HOPE'	`08MF005′	49.39	−121.45	26	−17.61	−135.53	−16.50	−124.57	−18.37	−142.43	−17.72	−136.58
`FRASER RIVER ABOVE TEXAS CREEK'	`08MF040′	50.61	−121.85	12	−18.07	−139.40	−17.35	−134.58	−19.16	−146.51	−18.18	−140.43
`FRASER RIVER AT MISSION'	`08MH024′	49.13	−122.30	29	−17.08	−130.36	−15.55	−115.20	−18.13	−137.87	n.d.	n.d.
`COLUMBIA RIVER AT DONALD'	`08NB005′	51.48	−117.18	28	−19.14	−146.20	−18.43	−140.12	−20.49	−154.46	−19.33	−147.01
`COLUMBIA RIVER AT BIRCHBANK'	`08NE049′	49.18	−117.72	24	−18.16	−137.14	−17.63	−132.84	−18.73	−140.35	−18.12	−136.78
`KOOTENAY RIVER AT FORT STEELE'	`08NG065′	49.61	−115.64	21	−18.36	−139.19	−17.10	−130.29	−18.90	−142.10	−18.45	−139.44
`LINE CREEK AT THE MOUTH'	`08NK022′	49.89	−114.83	1	−18.45	−139.29	−18.45	−139.29	−18.45	−139.29	n.d.	n.d.
`MISSION CREEK NEAR EAST KELOWNA'	`08NM116′	49.88	−119.41	12	−16.47	−126.77	−14.88	−117.96	−17.56	−132.87	−17.01	−130.70
`WHITEMAN CREEK ABOVE BOULEAU CREEK'	`08NM174′	50.21	−119.54	9	−16.73	−128.45	−16.31	−126.62	−16.96	−131.02	n.d.	n.d.
`LIARD RIVER AT LOWER CROSSING'	`10BE001′	59.41	−126.10	28	−21.14	−165.33	−20.18	−158.80	−23.39	−180.39	−21.09	−165.11
`FONTAS RIVER NEAR THE MOUTH'	`10CA001′	58.27	−121.46	17	−18.52	−150.72	−17.01	−141.26	−20.75	−163.92	−18.21	−148.29
`SIKANNI CHIEF RIVER NEAR FORT NELSON'	`10CB001′	57.24	−122.69	22	−20.46	−159.40	−18.80	−153.00	−21.02	−162.81	−20.75	−160.82

Note: n.d. = not determined; Mean=arithmetic mean, Max.=Maximum δ values, Min.=minimum δ value, Flow-weighted = weighted by discharge.

**Table 2:** Summary of isotope monitoring data for Alberta.

					Mean		Max.		Min.		Flow weighted	
Station Name	Station ID	Latitude	Longitude	Samples	δ^18^O	δ^2^H	δ^18^O	δ^2^H	δ^18^O	δ^2^H	δ^18^O	δ^2^H
`OLDMAN RIVER NEAR THE MOUTH'	`05AG006′	49.92	−111.80	37	−16.60	−129.44	−14.34	−122.22	−17.75	−136.74	−16.98	−131.41
`SOUTH SASKATCHEWAN RIVER AT MEDICINE HAT'	`05AJ001′	50.04	−110.68	23	−17.62	−137.59	−15.19	−124.64	−18.76	−147.36	−17.76	−138.75
`BOW RIVER AT CALGARY'	`05BH004′	51.05	−114.05	9	−18.98	−145.95	−18.71	−144.51	−19.51	−149.58	n.d.	n.d.
`BOW RIVER BELOW CARSELAND DAM'	`05BM002′	50.82	−113.44	15	−18.73	−144.86	−18.48	−142.65	−19.27	−148.28	−18.84	−145.41
`BOW RIVER BELOW BASSANO DAM'	`05BM004′	50.75	−112.54	12	−18.52	−143.59	−17.87	−139.95	−19.17	−148.52	−18.66	−144.61
`BOW RIVER NEAR THE MOUTH'	`05BN012′	50.05	−111.59	43	−18.15	−141.28	−16.21	−129.93	−20.21	−157.29	−18.26	−141.47
`LITTLE RED DEER RIVER NEAR WATER VALLEY'	`05CB002′	51.51	−114.67	1	−10.94	−98.52	−10.94	−98.52	−10.94	−98.52	n.d.	n.d.
`RED DEER RIVER AT DRUMHELLER'	`05CE001′	51.47	−112.71	27	−17.81	−140.96	−16.72	−134.66	−18.79	−147.08	−17.70	−140.05
`BLOOD INDIAN CREEK NEAR THE MOUTH'	`05CK001′	50.96	−111.06	1	−18.25	−143.50	−18.25	−143.50	−18.25	−143.50	n.d.	n.d.
`RED DEER RIVER NEAR BINDLOSS'	`05CK004′	50.90	−110.30	21	−17.56	−140.34	−15.40	−128.00	−20.46	−162.04	−17.52	−139.56
`NORTH SASKATCHEWAN RIVER AT WHIRLPOOL POINT'	`05DA009′	52.00	−116.47	2	−21.45	−162.50	−21.41	−162.00	−21.50	−163.00	n.d.	n.d.
`NORTH SASKATCHEWAN RIVER NEAR ROCKY MOUNTAIN HOUSE'	`05DC001′	52.38	−114.94	16	−19.53	−149.68	−18.77	−144.86	−20.24	−154.97	−19.37	−148.63
`NORTH SASKATCHEWAN RIVER NEAR LODGEPOLE(flow stop 1977)'	`05DE006′	53.05	−115.21	13	−19.52	−151.27	−19.14	−148.04	−19.84	−155.48	n.d.	n.d.
`NORTH SASKATCHEWAN RIVER AT HIGHWAY NO. 759'	`05DE010′	53.32	−114.76	31	−19.21	−148.71	−17.91	−140.31	−19.89	−155.30	−19.27	−149.21
`NORTH SASKATCHEWAN RIVER AT EDMONTON'	`05DF001′	53.54	−113.49	32	−19.39	−150.46	−18.49	−143.52	−21.09	−164.98	−19.20	−148.88
`PAINTEARTH CREEK NEAR HALKIRK'	`05FC004′	52.39	−112.14	1	−16.29	−139.07	−16.29	−139.07	−16.29	−139.07	n.d.	n.d.
`BATTLE RIVER AT HIGHWAY NO. 872'	`05FC008′	52.40	−111.41	16	−12.66	−119.69	−10.03	−106.84	−18.59	−152.66	−16.79	−142.45
`BATTLE RIVER NEAR THE SASKATCHEWAN BOUNDARY'	`05FE004′	52.86	−110.02	8	−15.08	−131.06	−12.48	−117.62	−18.34	−152.20	n.d.	n.d.
`BEAVER RIVER AT COLD LAKE RESERVE'	`06AD006′	54.36	−110.22	12	−14.37	−121.92	−13.41	−116.70	−15.43	−129.45	−14.40	−122.40
`ATHABASCA RIVER NEAR JASPER'	`07AA002′	52.91	−118.06	3	−19.99	−152.12	−19.89	−151.34	−20.13	−153.51	n.d.	n.d.
`ATHABASCA RIVER AT HINTON'	`07AD002′	53.42	−117.57	49	−19.96	−153.76	−19.15	−147.56	−21.18	−163.19	−20.35	−156.47
`PEMBINA RIVER AT JARVIE'	`07BC002′	54.45	−113.99	26	−16.85	−136.72	−15.23	−124.42	−19.83	−156.00	−16.47	−133.14
`ATHABASCA RIVER AT ATHABASCA'	`07BE001′	54.72	−113.29	33	−18.35	−144.95	−17.01	−137.01	−19.92	−160.16	−18.35	−144.07
`EAST PRAIRIE RIVER NEAR ENILDA'	`07BF001′	55.42	−116.34	1	−19.13	−148.97	−19.13	−148.97	−19.13	−148.97	n.d.	n.d.
`SWAN RIVER NEAR KINUSO'	`07BJ001′	55.32	−115.42	5	−17.36	−135.81	−16.91	−131.54	−17.81	−139.00	n.d.	n.d.
`LESSER SLAVE RIVER AT SLAVE LAKE'	`07BK001′	55.30	−114.76	25	−13.53	−118.01	−13.06	−114.63	−14.47	−123.60	−13.68	−118.95
`CLEARWATER RIVER AT DRAPER'	`07CD001′	56.69	−111.26	9	−16.00	−130.54	−15.59	−124.99	−17.68	−137.38	n.d.	n.d.
`HANGINGSTONE RIVER AT FORT MCMURRAY'	`07CD004′	56.71	−111.36	1	−16.53	−131.63	−16.53	−131.63	−16.53	−131.63	n.d.	n.d.
`CLEARWATER RIVER ABOVE CHRISTINA RIVER'	`07CD005′	56.66	−110.93	6	−15.97	−131.63	−15.44	−128.97	−17.15	−135.48	n.d.	n.d.
`GREGOIRE LAKE NEAR FORT MCMURRAY'	`07CE001′	56.48	−111.18	2	−19.77	−154.21	−18.85	−147.40	−20.70	−161.03	n.d.	n.d.
`CHRISTINA RIVER NEAR CHARD'	`07CE002′	55.84	−110.87	14	−16.25	−131.19	−14.83	−122.24	−18.87	−150.13	−16.68	−132.99
`PONY CREEK NEAR CHARD'	`07CE003′	55.87	−110.92	1	−15.53	−123.48	−15.53	−123.48	−15.53	−123.48	n.d.	n.d.
`ATHABASCA RIVER BELOW FORT MCMURRAY'	`07DA001′	56.78	−111.40	7	−17.36	−139.49	−15.27	−129.07	−18.19	−143.24	n.d.	n.d.
`MACKAY RIVER NEAR FORT MACKAY'	`07DB001′	57.21	−111.70	1	−14.63	−129.03	−14.63	−129.03	−14.63	−129.03	n.d.	n.d.
`FIREBAG RIVER NEAR THE MOUTH'	`07DC001′	57.65	−111.20	16	−17.80	−142.03	−17.03	−137.23	−18.37	−147.30	−17.91	−142.08
`PEACE RIVER AT DUNVEGAN BRIDGE'	`07FD003′	55.92	−118.61	13	−19.17	−149.19	−18.82	−146.57	−19.53	−151.30	−19.16	−149.20
`EUREKA RIVER NEAR WORSLEY'	`07FD013′	56.45	−119.13	1	−19.83	−153.93	−19.83	−153.93	−19.83	−153.93	n.d.	n.d.
`WAPITI RIVER NEAR GRANDE PRAIRIE'	`07GE001′	55.07	−118.80	26	−19.16	−148.20	−18.25	−140.14	−21.07	−165.55	−19.75	−151.99
`LITTLE SMOKY RIVER NEAR GUY'	`07GH002′	55.46	−117.16	23	−17.88	−143.62	−15.75	−131.96	−19.74	−157.96	−18.16	−144.99
`SMOKY RIVER AT WATINO'	`07GJ001′	55.71	−117.62	16	−18.79	−148.16	−16.72	−136.34	−20.51	−162.84	−18.96	−148.76
`PEACE RIVER AT PEACE RIVER'	`07HA001′	56.24	−117.31	16	−19.30	−149.91	−18.69	−145.61	−20.46	−157.09	−19.35	−150.16
`NOTIKEWIN RIVER AT MANNING'	`07HC001′	56.92	−117.62	1	−17.76	−147.68	−17.76	−147.68	−17.76	−147.68	n.d.	n.d.
`PEACE RIVER NEAR CARCAJOU(Frozen 1967)'	`07HD001′	57.74	−117.03	24	−19.39	−150.42	−18.71	−145.25	−20.82	−161.49	n.d.	n.d.
`WABASCA RIVER AT HIGHWAY NO. 88'	`07JD002′	57.87	−115.39	31	−15.09	−128.52	−13.28	−120.73	−18.20	−146.42	−16.57	−136.43
`PEACE RIVER AT PEACE POINT (ALBERTA)'	`07KC001′	59.12	−112.44	18	−19.61	−153.12	−16.75	−135.26	−23.21	−181.50	−19.44	−152.09
`BIRCH RIVER BELOW ALICE CREEK'	`07KE001′	58.32	−113.07	8	−17.56	−142.31	−15.05	−132.01	−19.51	−153.66	n.d.	n.d.
`SLAVE RIVER AT FITZGERALD (ALBERTA)'	`07NB001′	59.87	−111.58	12	−18.90	−149.50	−16.90	−136.97	−22.55	−173.83	−18.21	−144.79
`HAY RIVER NEAR MEANDER RIVER'	`07OB003′	59.15	−117.64	24	−17.06	−143.21	−13.85	−125.63	−20.74	−163.79	−18.09	−147.17
`CHINCHAGA RIVER NEAR HIGH LEVEL'	`07OC001′	58.60	−118.33	32	−18.10	−148.01	−15.45	−135.24	−21.72	−169.92	−19.25	−153.79

Note: n.d. = not determined; Mean=arithmetic mean, Max.=Maximum δ values, Min.=minimum δ value, Flow-weighted = weighted by discharge.

**Table 3:** Summary of isotope monitoring data for Saskatchewan.

					Mean		Max.		Min.		Flow weighted	
Station Name	Station ID	Latitude	Longitude	Samples	δ^18^O	δ^2^H	δ^18^O	δ^2^H	δ^18^O	δ^2^H	δ^18^O	δ^2^H
`NORTH SASKATCHEWAN RIVER NEAR DEER CREEK'	`05EF001′	53.52	−109.62	5	−18.90	−147.35	−18.21	−143.09	−19.30	−149.96	n.d.	n.d.
`NORTH SASKATCHEWAN RIVER AT PRINCE ALBERT'	`05GG001′	53.20	−105.77	5	−17.63	−139.76	−16.48	−133.26	−18.20	−143.66	n.d.	n.d.
`SOUTH SASKATCHEWAN RIVER AT SASKATOON'	`05HG001′	52.14	−106.64	4	−17.19	−136.02	−16.90	−135.23	−17.36	−136.91	n.d.	n.d.
`MOOSE JAW RIVER NEAR BURDICK'	`05JE006′	50.40	−105.40	7	−11.95	−98.47	−10.19	−82.74	−14.64	−116.12	n.d.	n.d.
`QUAPPELLE RIVER NEAR LUMSDEN'	`05JF001′	50.65	−104.87	9	−12.75	−107.34	−10.23	−85.75	−15.45	−127.68	n.d.	n.d.
`WASCANA CREEK NEAR LUMSDEN'	`05JF005′	50.64	−104.91	8	−13.14	−110.13	−12.04	−102.03	−14.80	−124.52	n.d.	n.d.
`QUAPPELLE RIVER BELOW CRAVEN DAM'	`05JK002′	50.71	−104.80	4	−11.22	−99.15	−10.15	−93.75	−12.50	−106.04	n.d.	n.d.
`QUAPPELLE RIVER NEAR WELBY'	`05JM001′	50.49	−101.56	16	−13.02	−109.55	−10.57	−92.43	−19.28	−153.11	−13.65	−114.98
`SASKATCHEWAN RIVER BELOW TOBIN LAKE'	`05KD003′	53.71	−103.30	4	−16.87	−134.90	−16.40	−132.10	−17.58	−138.46	n.d.	n.d.
`CARROT RIVER NEAR TURNBERRY'	`05KH007′	53.61	−102.10	18	−15.25	−121.91	−11.24	−100.00	−19.75	−154.11	−16.18	−127.37
`RED DEER RIVER NEAR ERWOOD'	`05LC001′	52.86	−102.20	12	−14.51	−116.40	−11.74	−102.25	−20.35	−154.49	−15.59	−123.39
`WHITESAND RIVER NEAR CANORA'	`05MB003′	51.64	−102.37	13	−12.39	−104.50	−8.62	−85.09	−18.83	−146.19	n.d.	n.d.
`WHITESAND RIVER NEAR SHEHO'	`05MB004′	51.59	−103.04	2	−18.49	−142.76	−18.38	−141.83	−18.59	−143.69	n.d.	n.d.
`ASSINIBOINE RIVER AT STURGIS'	`05MC001′	51.94	−102.55	15	−13.36	−110.16	−11.28	−95.59	−20.18	−158.44	−14.70	−120.21
`ASSINIBOINE RIVER AT KAMSACK'	`05MD004′	51.56	−101.92	14	−12.84	−108.07	−9.73	−89.94	−19.20	−148.54	−16.07	−128.04
`LONG CREEK NEAR ESTEVAN'	`05NB001′	49.10	−103.02	8	−12.08	−102.82	−9.14	−89.63	−16.09	−129.46	n.d.	n.d.
`SOURIS RIVER BELOW RAFFERTY RESERVOIR'	`05NB036′	49.14	−103.08	8	−10.12	−87.66	−9.61	−82.04	−10.68	−89.59	n.d.	n.d.
`MOOSE MOUNTAIN CREEK NEAR OXBOW'	`05ND004′	49.23	−102.23	7	−11.90	−101.94	−11.04	−97.45	−13.75	−113.92	n.d.	n.d.
`MOOSE MOUNTAIN CREEK ABOVE ALAMEDA RESERVOIR'	`05ND010′	49.52	−102.17	9	−12.06	−100.87	−10.56	−86.19	−14.05	−111.92	n.d.	n.d.
`RABBIT CREEK NEAR MAKWA'	`06AD005′	54.06	−108.85	1	−15.23	−125.95	−15.23	−125.95	−15.23	−125.95	n.d.	n.d.
`RAPID RIVER AT OUTLET OF LAC LA RONGE'	`06CB002′	55.35	−104.50	12	−11.32	−100.51	−10.93	−98.45	−13.43	−114.52	−11.19	−99.46
`CHURCHILL RIVER ABOVE OTTER RAPIDS'	`06CD002′	55.65	−104.76	9	−13.13	−113.67	−12.56	−110.77	−13.59	−115.42	n.d.	n.d.
`GEIKIE RIVER BELOW WHEELER RIVER'	`06DA004′	57.58	−104.19	13	−14.81	−125.75	−13.64	−120.38	−15.92	−131.58	−15.00	−126.54
`WATHAMAN RIVER BELOW WATHAMAN LAKE'	`06DC001′	57.09	−103.71	11	−14.03	−119.90	−13.14	−113.91	−14.93	−126.35	−14.26	−121.69
`REINDEER RIVER ABOVE DEVIL RAPIDS'	`06DD002′	56.19	−103.16	8	−13.18	−113.28	−11.01	−98.68	−13.91	−117.80	n.d.	n.d.
`CHURCHILL RIVER AT SANDY BAY'	`06EA002′	55.52	−102.32	8	−12.92	−112.08	−12.75	−110.89	−13.48	−115.14	n.d.	n.d.
`FOND DU LAC RIVER AT OUTLET OF BLACK LAKE'	`07LE002′	59.15	−105.54	11	−15.72	−131.30	−15.27	−129.05	−16.13	−133.60	−15.70	−131.11
`MACFARLANE RIVER AT OUTLET OF DAVY LAKE'	`07MB001′	58.97	−108.17	9	−16.46	−136.51	−15.27	−130.65	−17.64	−142.99	n.d.	n.d.

Note: n.d. = not determined; Mean=arithmetic mean, Max.=Maximum δ values, Min.=minimum δ value, Flow-weighted = weighted by discharge.

**Table 4:** Summary of isotope monitoring data for Manitoba.

					Mean		Max.		Min.		Flow weighted	
Station Name	Station ID	Latitude	Longitude	Samples	δ^18^O	δ^2^H	δ^18^O	δ^2^H	δ^18^O	δ^2^H	δ^18^O	δ^2^H
`HAYES RIVER BELOW GODS RIVER'	`04AB001′	56.42	−92.81	11	−12.76	−104.51	−11.57	−97.27	−15.18	−118.70	−13.68	−109.96
`GODS RIVER NEAR SHAMATTAWA'	`04AD002′	55.85	−92.09	17	−11.94	−99.32	−11.22	−94.81	−13.05	−106.21	−12.09	−100.28
`SASKATCHEWAN RIVER AT THE PAS'	`05KJ001′	53.84	−101.21	29	−15.79	−126.85	−11.29	−99.29	−18.31	−144.16	−16.12	−128.75
`WATERHEN RIVER NEAR WATERHEN'	`05LH005′	51.85	−99.55	10	−9.64	−84.56	−8.89	−80.79	−10.33	−88.81	−9.67	−84.56
`VALLEY RIVER NEAR DAUPHIN'	`05LJ010′	51.28	−100.02	16	−12.07	−99.62	−9.30	−85.44	−16.28	−121.87	−13.96	−110.97
`MOSSY RIVER BELOW OUTLET OF DAUPHIN LAKE'	`05LJ025′	51.45	−99.97	24	−10.99	−91.15	−9.28	−80.91	−13.50	−106.38	−11.13	−92.02
`WHITEMUD RIVER NEAR KEYES'	`05LL005′	50.19	−99.11	16	−12.71	−101.84	−8.60	−82.18	−17.14	−131.92	−13.68	−107.84
`LAKE MANITOBA NEAR WESTBOURNE'	`05LL012′	50.25	−98.59	5	−11.96	−95.04	−9.83	−82.71	−15.22	−116.03	n.d.	n.d.
`FAIRFORD RIVER NEAR FAIRFORD'	`05LM001′	51.59	−98.71	19	−8.60	−76.50	−7.96	−72.06	−9.22	−79.42	−8.67	−76.80
`LAKE ST. MARTIN NEAR HILBRE'	`05LM005′	51.51	−98.53	1	−8.29	−75.14	−8.29	−75.14	−8.29	−75.14	n.d.	n.d.
`DAUPHIN RIVER NEAR DAUPHIN RIVER'	`05LM006′	52.00	−98.33	21	−8.93	−78.52	−7.88	−71.02	−10.09	−87.28	−9.16	−79.97
`ASSINIBOINE RIVER NEAR RUSSELL'	`05ME001′	50.81	−101.44	9	−13.82	−113.39	−11.28	−95.09	−16.10	−128.61	n.d.	n.d.
`ASSINIBOINE RIVER NEAR MINIOTA'	`05ME006′	50.11	−101.04	21	−13.17	−109.05	−11.42	−91.84	−19.34	−150.17	−13.61	−110.49
`ASSINIBOINE RIVER WEST OF RUSSELL'	`05ME012′	50.77	−101.44	10	−13.55	−111.65	−11.91	−96.60	−15.84	−124.97	−14.42	−116.26
`ASSINIBOINE RIVER AT BRANDON'	`05MH001′	49.86	−99.96	3	−12.21	−103.05	−11.99	−101.33	−12.55	−105.06	n.d.	n.d.
`ASSINIBOINE RIVER NEAR HOLLAND'	`05MH005′	49.70	−98.89	29	−12.55	−101.60	−10.44	−86.62	−16.91	−131.95	−13.13	−102.87
`EPINETTE CREEK NEAR CARBERRY'	`05MH007′	49.74	−99.33	1	−13.48	−110.48	−13.48	−110.48	−13.48	−110.48	n.d.	n.d.
`ASSINIBOINE RIVER NEAR BRANDON'	`05MH013′	49.87	−100.10	8	−11.91	−101.42	−11.18	−93.64	−14.06	−115.97	n.d.	n.d.
`ASSINIBOINE RIVER AT HEADINGLEY'	`05MJ001′	49.87	−97.41	12	−11.68	−97.26	−10.57	−87.70	−14.75	−118.95	−11.84	−97.61
`SOURIS RIVER AT MELITA'	`05NF001′	49.27	−100.98	16	−8.20	−77.10	−4.31	−57.16	−13.52	−107.08	−11.16	−91.04
`ANTLER RIVER NEAR MELITA'	`05NF002′	49.06	−101.05	17	−9.70	−86.66	−6.95	−69.15	−14.52	−113.47	−11.19	−94.77
`SOURIS RIVER AT WAWANESA'	`05NG001′	49.60	−99.68	8	−8.40	−77.77	−6.21	−64.12	−12.74	−105.06	n.d.	n.d.
`OAK CREEK NEAR STOCKTON'	`05NG010′	49.56	−99.51	12	−11.87	−96.89	−6.13	−57.58	−18.81	−140.78	−9.46	−80.86
`PEMBINA RIVER ABOVE LORNE LAKE'	`05OA010′	49.26	−99.46	29	−12.63	−100.19	−8.50	−66.12	−20.78	−160.27	−15.93	−123.73
`RED RIVER AT EMERSON'	`05OC001′	49.01	−97.22	18	−9.01	−71.43	−6.58	−48.65	−16.86	−127.22	−10.70	−81.53
`RED RIVER NEAR ST. NORBERT'	`05OC008′	49.79	−97.13	30	−9.87	−77.43	−6.42	−50.13	−17.44	−132.41	−11.77	−89.26
`RED RIVER NEAR STE. AGATHE'	`05OC012′	49.55	−97.19	14	−9.32	−73.01	−6.77	−50.50	−17.43	−131.19	−11.49	−87.36
`TOBACCO CREEK NEAR ROSEBANK'	`05OF018′	49.43	−98.16	15	−13.30	−102.05	−10.71	−80.98	−18.80	−146.20	−13.02	−95.79
`LA SALLE RIVER NEAR SANFORD'	`05OG001′	49.68	−97.43	1	−14.88	−115.56	−14.88	−115.56	−14.88	−115.56	n.d.	n.d.
`RED RIVER AT SELKIRK'	`05OJ005′	50.15	−96.87	19	−10.91	−86.92	−7.32	−64.77	−15.27	−114.92	−10.39	−82.32
`WHITESHELL RIVER AT OUTLET OF JESSICA LAKE'	`05PG001′	50.03	−95.51	19	−8.70	−74.44	−7.40	−63.81	−10.73	−88.10	−8.77	−73.86
`BIRD RIVER AT OUTLET OF BIRD LAKE'	`05PJ001′	50.46	−95.41	1	−8.89	−75.02	−8.89	−75.02	−8.89	−75.02	n.d.	n.d.
`GRASS RIVER ABOVE STANDING STONE FALLS'	`05TD001′	55.74	−97.01	8	−12.46	−104.61	−11.81	−100.47	−13.08	−108.32	n.d.	n.d.
`FOOTPRINT LAKE AT NELSON HOUSE'	`05TF001′	55.78	−98.88	2	−12.73	−109.54	−12.67	−107.48	−12.79	−111.60	n.d.	n.d.
`FOOTPRINT RIVER ABOVE FOOTPRINT LAKE'	`05TF002′	55.93	−98.89	2	−12.74	−108.28	−12.27	−104.53	−13.22	−112.03	n.d.	n.d.
`BURNTWOOD RIVER NEAR THOMPSON'	`05TG001′	55.74	−97.90	25	−12.88	−111.96	−9.58	−109.84	−13.99	−116.55	−12.90	−112.21
`TAYLOR RIVER NEAR THOMPSON'	`05TG002′	55.49	−98.19	2	−14.47	−115.70	−14.06	−114.53	−14.88	−116.88	n.d.	n.d.
`ODEI RIVER NEAR THOMPSON'	`05TG003′	56.00	−97.36	9	−14.68	−116.40	−13.50	−109.47	−16.29	−128.85	n.d.	n.d.
`SAPOCHI RIVER NEAR NELSON HOUSE'	`05TG006′	55.91	−98.49	8	−15.60	−121.28	−14.09	−110.64	−17.36	−135.68	n.d.	n.d.
`SPLIT LAKE AT SPLIT LAKE'	`05UF003′	56.24	−96.09	16	−11.90	−99.87	−11.02	−93.80	−13.68	−110.08	n.d.	n.d.
`LIMESTONE RIVER NEAR BIRD'	`05UG001′	56.51	−94.22	22	−14.72	−116.59	−12.01	−105.97	−18.28	−137.79	−14.54	−113.93
`COCHRANE RIVER NEAR BROCHET'	`06DA002′	58.00	−101.40	14	−14.17	−120.88	−13.85	−118.57	−14.56	−123.46	−14.20	−120.90
`CHURCHILL RIVER ABOVE GRANVILLE FALLS'	`06EA006′	56.15	−100.46	10	−13.06	−113.06	−12.72	−110.54	−14.06	−118.45	−13.17	−113.60
`CHURCHILL RIVER ABOVE LEAF RAPIDS'	`06EB004′	56.47	−100.04	14	−13.01	−112.05	−11.86	−102.63	−13.92	−118.26	−13.17	−113.12
`SOUTH BAY DIVERSION CHANNEL AT SOUTH BAY'	`06EC002′	56.67	−99.04	14	−12.84	−111.17	−12.39	−107.55	−13.40	−115.54	−12.78	−110.53
`CHURCHILL RIVER BELOW FIDLER LAKE'	`06FB001′	57.24	−96.83	14	−13.53	−113.31	−12.81	−110.25	−14.47	−117.44	−13.40	−112.60
`LITTLE CHURCHILL RIVER ABOVE RECLUSE LAKE'	`06FC001′	56.95	−95.74	4	−14.71	−116.93	−14.12	−114.20	−15.45	−120.21	n.d.	n.d.
`CHURCHILL RIVER ABOVE RED HEAD RAPIDS'	`06FD001′	58.12	−94.62	18	−14.30	−117.17	−12.77	−107.41	−21.75	−164.89	−13.83	−114.02
`SEAL RIVER BELOW GREAT ISLAND'	`06GD001′	58.89	−96.28	14	−15.21	−122.97	−14.33	−117.22	−16.60	−132.01	−14.92	−121.00

Note: n.d. = not determined; Mean=arithmetic mean, Max.=Maximum δ values, Min.=minimum δ value, Flow-weighted = weighted by discharge.

**Table 5:** Summary of isotope monitoring data for Ontario.

					Mean		Max.		Min.		Flow weighted	
Station Name	Station ID	Latitude	Longitude	Samples	δ^18^O	δ^2^H	δ^18^O	δ^2^H	δ^18^O	δ^2^H	δ^18^O	δ^2^H
`KAMINISTIQUIA RIVER AT KAMINISTIQUIA'	`02AB006′	48.53	−89.60	1	−9.83	−70.38	−9.83	−70.38	−9.83	−70.38	n.d.	n.d.
`GRAVEL RIVER NEAR CAVERS'	`02AE001′	48.93	−87.69	1	−11.10	−84.18	−11.10	−84.18	−11.10	−84.18	n.d.	n.d.
`PIC RIVER NEAR MARATHON'	`02BB003′	48.77	−86.30	1	−11.06	−83.46	−11.06	−83.46	−11.06	−83.46	n.d.	n.d.
`FRENCH RIVER AT DRY PINE BAY'	`02DD010′	46.07	−80.61	12	−9.40	−70.47	−6.89	−60.23	−11.20	−79.56	−10.06	−73.32
`MAGNETAWAN RIVER NEAR BRITT'	`02EA011′	45.77	−80.48	13	−9.89	−72.30	−9.08	−64.84	−11.01	−77.42	−9.81	−71.63
`BLACK RIVER NEAR WASHAGO'	`02EC002′	44.71	−79.28	9	−9.00	−64.23	−7.25	−55.03	−9.94	−70.38	n.d.	n.d.
`NORTH PENETANGORE RIVER AT KINCARDINE'	`02FD003′	44.17	−81.63	1	−19.95	−154.01	−19.95	−154.01	−19.95	−154.01	n.d.	n.d.
`GRAND RIVER AT BRANTFORD'	`02GB001′	43.13	−80.27	12	−9.44	−64.19	−8.52	−57.48	−10.71	−70.44	−9.61	−64.56
`THAMES RIVER AT THAMESVILLE'	`02GE003′	42.54	−81.97	18	−8.72	−59.01	−7.54	−51.84	−10.14	−67.83	−9.19	−60.89
`SYDENHAM RIVER NEAR ALVINSTON'	`02GG002′	42.83	−81.85	18	−9.30	−62.05	−7.86	−52.19	−10.16	−68.32	−9.46	−62.30
`SYDENHAM RIVER AT FLORENCE'	`02GG003′	42.65	−82.01	1	−10.58	−68.32	−10.58	−68.32	−10.58	−68.32	n.d.	n.d.
`NIAGARA RIVER AT FORT ERIE'	`02HA013′	42.93	−78.91	7	−6.44	−48.66	−6.30	−47.12	−6.65	−50.57	n.d.	n.d.
`LYNDE CREEK NEAR WHITBY'	`02HC018′	43.88	−78.96	5	−9.61	−65.98	−8.91	−61.10	−10.58	−70.14	n.d.	n.d.
`TRENT RIVER AT TRENTON'	`02HK010′	44.12	−77.59	4	−8.74	−62.65	−7.78	−57.85	−9.81	−68.14	n.d.	n.d.
`YORK RIVER NEAR BANCROFT'	`02KD002′	45.05	−77.85	24	−9.19	−66.32	−6.86	−59.42	−10.59	−74.22	−9.53	−68.03
`OTTAWA RIVER AT BRITANNIA'	`02KF005′	45.35	−75.83	27	−10.63	−78.22	−9.58	−71.48	−11.56	−84.07	−10.79	−78.94
`SEVERN RIVER AT OUTLET OF MUSKRAT DAM LAKE'	`04CA002′	53.49	−91.51	8	−12.62	−101.28	−11.38	−92.73	−14.87	−116.72	n.d.	n.d.
`WINDIGO RIVER ABOVE MUSKRAT DAM LAKE'	`04CB001′	53.35	−91.79	8	−13.01	−102.52	−11.13	−91.95	−15.33	−118.30	n.d.	n.d.
`PIPESTONE RIVER AT KARL LAKE'	`04DA001′	52.58	−90.19	7	−12.86	−101.01	−11.51	−89.25	−15.67	−119.22	n.d.	n.d.
`ASHEWEIG RIVER AT STRAIGHT LAKE'	`04DB001′	53.71	−87.95	11	−13.21	−105.54	−11.85	−96.42	−15.09	−117.59	−13.96	−110.24
`WINISK RIVER BELOW ASHEWEIG RIVER TRIBUTARY'	`04DC001′	54.50	−87.23	10	−12.89	−102.31	−11.60	−92.54	−14.82	−114.76	n.d.	n.d.
`OTOSKWIN RIVER BELOW BADESDAWA LAKE'	`04FA001′	51.82	−89.60	4	−11.28	−89.42	−10.77	−84.75	−12.65	−98.12	n.d.	n.d.
`ATTAWAPISKAT RIVER BELOW MUKETEI RIVER'	`04FC001′	53.09	−85.07	9	−13.57	−104.45	−11.30	−91.67	−16.11	−121.25	n.d.	n.d.
`CAT RIVER BELOW WESLEYAN LAKE'	`04GA002′	51.17	−91.59	4	−11.76	−95.23	−10.75	−90.04	−12.76	−101.85	n.d.	n.d.
`OGOKI RIVER ABOVE WHITECLAY LAKE'	`04GB004′	50.87	−88.93	8	−11.89	−92.97	−10.56	−84.51	−12.64	−99.34	n.d.	n.d.
`ALBANY RIVER BELOW ACHAPI LAKE'	`04GC002′	51.37	−89.42	5	−11.76	−92.80	−10.50	−86.45	−12.61	−97.04	n.d.	n.d.
`ALBANY RIVER NEAR HAT ISLAND'	`04HA001′	51.33	−83.83	13	−12.64	−97.24	−10.86	−86.45	−15.08	−113.29	−13.69	−103.46
`LITTLE CURRENT RIVER AT PERCY LAKE'	`04JF001′	50.66	−86.52	9	−12.70	−98.04	−10.86	−86.89	−15.04	−112.19	n.d.	n.d.
`KENOGAMI RIVER NEAR MAMMAMATTAWA'	`04JG001′	50.42	−84.38	14	−12.51	−93.09	−10.91	−83.24	−15.03	−110.84	−13.72	−101.12
`MATTAGAMI RIVER NEAR TIMMINS'	`04LA002′	48.40	−81.45	6	−12.07	−90.60	−10.71	−82.46	−14.39	−104.58	n.d.	n.d.
`GROUNDHOG RIVER AT FAUQUIER'	`04LD001′	49.31	−82.04	20	−11.75	−86.92	−10.15	−76.50	−15.63	−112.73	−12.93	−94.28
`KAPUSKASING RIVER AT KAPUSKASING'	`04LF001′	49.41	−82.44	20	−12.03	−88.84	−10.07	−75.83	−15.98	−115.32	−13.77	−99.95
`MOOSE RIVER ABOVE MOOSE RIVER'	`04LG004′	50.75	−81.45	7	−12.78	−94.18	−10.48	−80.01	−14.96	−108.67	n.d.	n.d.
`MISSINAIBI RIVER AT MATTICE'	`04LJ001′	49.61	−83.27	16	−12.18	−90.38	−10.29	−79.36	−16.32	−117.35	−14.41	−104.68
`MISSINAIBI RIVER BELOW WABOOSE RIVER'	`04LM001′	50.59	−82.09	6	−12.66	−94.02	−10.16	−80.07	−14.86	−108.98	n.d.	n.d.
`ABITIBI RIVER AT ONAKAWANA'	`04ME003′	50.60	−81.41	6	−12.76	−93.91	−10.98	−83.17	−14.89	−107.87	n.d.	n.d.
`NORTH FRENCH RIVER NEAR THE MOUTH'	`04MF001′	51.08	−80.76	5	−13.27	−97.84	−11.05	−85.72	−15.03	−110.56	n.d.	n.d.
`NAMAKAN RIVER AT OUTLET OF LAC LA CROIX'	`05PA006′	48.38	−92.18	2	−7.83	−66.48	−7.69	−65.44	−7.96	−67.52	n.d.	n.d.
`ATIKOKAN RIVER AT ATIKOKAN'	`05PB018′	48.75	−91.58	7	−8.65	−72.08	−7.97	−68.30	−9.36	−76.30	n.d.	n.d.
`ENGLISH RIVER AT UMFREVILLE'	`05QA002′	49.87	−91.46	3	−12.27	−94.53	−11.14	−88.33	−14.43	−106.79	n.d.	n.d.
`WABIGOON RIVER NEAR QUIBELL'	`05QD006′	49.96	−93.40	1	−8.42	−68.02	−8.42	−68.02	−8.42	−68.02	n.d.	n.d.

Note: n.d. = not determined; Mean=arithmetic mean, Max.=Maximum δ values, Min.=minimum δ value, Flow-weighted = weighted by discharge.

**Table 6:** Summary of isotope monitoring data for Quebec.

											Flow	
					Mean		Max.		Min.		weighted	
Station Name	Station ID	Latitude	Longitude	Samples	δ^18^O	δ^2^H	δ^18^O	δ^2^H	δ^18^O	δ^2^H	δ^18^O	δ^2^H
`OUTAOUAIS (RIVIERE DES) A LA SORTIE DU LAC GRANET'	`02JB009′	47.84	−77.55	1	−12.32	−89.19	−12.32	−89.19	−12.32	−89.19	n.d.	n.d.
`KINOJEVIS (RIVIERE) A 0,3 KM EN AMONT DU PONT-ROUTE A CLÉRICY'	`02JB013′	48.37	−78.85	8	−11.38	−84.39	−10.51	−76.31	−12.07	−88.51	n.d.	n.d.
`MASKINONGE (RIVIERE) AU PONT DU C.N. PRÈS DE SAINTE-URSULE'	`02OC002′	46.30	−73.10	18	−10.38	−72.05	−9.35	−64.96	−11.20	−75.79	n.d.	n.d.
`RICHELIEU (RIVIERE) AUX RAPIDES FRYERS'	`02OJ007′	45.40	−73.26	1	−9.49	−66.20	−9.49	−66.20	−9.49	−66.20	n.d.	n.d.
`LOUP (RIVIERE DU) À 0,6 KM EN AMONT DU PONT-ROUTE 185′	`02PG001′	47.82	−69.52	15	−12.11	−87.02	−10.65	−79.03	−13.81	−101.39	n.d.	n.d.
`CHAUDIERE (RIVIERE) EN AVAL DU BARRAGE A MEGANTIC'	`02PJ012′	45.57	−70.88	1	−11.21	−80.06	−11.21	−80.06	−11.21	−80.06	n.d.	n.d.
`RIMOUSKI (RIVIERE) À 3,7 KM EN AMONT DU PONT-ROUTE 132′	`02QA002′	48.41	−68.56	15	−11.79	−83.63	−10.09	−73.46	−14.19	−100.14	n.d.	n.d.
`MISTASSIBI (RIVIÈRE) EN AMONT DE LA CHUTE BOUCHARD'	`02RD007′	48.94	−72.17	18	−13.48	−97.21	−12.38	−89.82	−15.74	−114.55	n.d.	n.d.
`ASHUAPMUSHAN (RIVIERE) À LA TÊTE DE LA CHUTE AUX SAUMONS'	`02RF001′	48.69	−72.49	14	−12.86	−93.82	−11.64	−85.55	−13.97	−103.08	n.d.	n.d.
`GODBOUT (RIVIERE) À 1,6 KM EN AMONT DU PONT-ROUTE 138′	`02UA003′	49.33	−67.66	9	−11.65	−84.63	−11.10	−81.18	−12.77	−92.53	n.d.	n.d.
`MOISIE (RIVIERE) À 5,1 KM EN AMONT DU PONT DU Q.N.S.L.R.'	`02UC002′	50.35	−66.19	5	−14.00	−103.13	−13.34	−98.44	−14.72	−107.90	n.d.	n.d.
`MAGPIE (RIVIERE) A LA SORTIE DU LAC MAGPIE'	`02VB004′	50.69	−64.58	1	−13.95	−101.91	−13.95	−101.91	−13.95	−101.91	n.d.	n.d.
`ROMAINE (RIVIERE) AU PONT DE LA Q.I.T.'	`02VC001′	50.31	−63.62	1	−14.17	−102.16	−14.17	−102.16	−14.17	−102.16	n.d.	n.d.
`NATASHQUAN (RIVIERE) À 0,6 KM EN AVAL DE LA DÉCHARGE DU LAC ALIESTE'	`02WB003′	50.43	−61.71	7	−13.49	−98.00	−12.44	−89.20	−15.02	−105.80	n.d.	n.d.
`PETIT MECATINA (RIVIERE DU) À 1,0 KM EN AMONT DE LA NÉTAGAMIOU'	`02XA008′	50.68	−59.59	6	−13.12	−95.34	−12.41	−90.74	−14.04	−100.76	n.d.	n.d.
`WASWANIPI (RIVIERE) A LA CHUTE ROUGE'	`03AB002′	49.86	−77.19	4	−13.20	−97.38	−12.31	−91.85	−15.42	−111.97	n.d.	n.d.
`BELL (RIVIERE) EN AMONT DU LAC MATAGAMI'	`03AC004′	49.75	−77.61	6	−12.12	−88.83	−11.23	−84.15	−12.92	−94.33	n.d.	n.d.
`PONTAX (RIVIERE) À 60,4 KM DE LEMBOUCHURE'	`03BF001′	51.53	−78.10	7	−13.56	−99.79	−11.36	−81.87	−14.39	−105.93	n.d.	n.d.
`BALEINE (GRANDE RIVIERE DE LA) EN AMONT DE LA RIVIERE DENYS-1′	`03ED001′	55.24	−76.98	5	−14.57	−111.20	−14.19	−109.81	−14.87	−113.21	n.d.	n.d.
`LOUPS MARINS (LAC DES) DANS LE BASSIN VERSANT DE LA RIVIÈRE NASTAPOCA'	`03FA003′	56.45	−74.24	4	−14.55	−112.52	−14.27	−111.99	−14.81	−113.54	n.d.	n.d.
`FEUILLES (RIVIERE AUX) EN AMONT DU RUISSEAU DUFREBOY'	`03JB004′	58.28	−71.28	5	−14.82	−113.74	−14.28	−109.68	−15.37	−118.97	n.d.	n.d.
`MELEZES (RIVIERE AUX) À 7,6 KM EN AMONT DE LA CONFLUENCE AVEC LA KOKSOAK'	`03KC004′	57.67	−69.61	7	−17.31	−130.82	−15.38	−117.58	−24.90	−189.50	n.d.	n.d.
`CANIAPISCAU (RIVIERE) À 1,0 KM EN AMONT DE LA CHUTE DE LA PYRITE'	`03LF002′	57.43	−69.21	6	−17.66	−133.59	−15.95	−120.50	−25.24	−192.38	n.d.	n.d.
`BALEINE (RIVIERE A LA) À 40,2 KM DE LEMBOUCHURE'	`03MB002′	57.88	−67.58	7	−17.01	−130.19	−15.09	−117.44	−24.79	−187.57	n.d.	n.d.
`GEORGE (RIVIERE) À LA SORTIE DU LAC DE LA HUTTE SAUVAGE'	`03MD001′	56.79	−65.76	4	−15.75	−119.21	−15.29	−114.51	−16.30	−123.50	n.d.	n.d.
BOURLAMAQUE (RIVIERE) 1.1KM FROM ROUTE 117	`04NA005′	48.09	−77.66	1	−14.25	−102.00	−14.25	−102.00	−14.25	−102.00	n.d.	n.d.

Note: n.d. = not determined; Mean=arithmetic mean, Max.=Maximum δ values, Min.=minimum δ value, Flow-weighted = weighted by discharge.

**Table 7:** Summary of isotope monitoring data for New Brunswick.

					Mean		Max.		Min.		Flow weighted	
Station Name	Station ID	Latitude	Longitude	Samples	δ^18^O	δ^2^H	δ^18^O	δ^2^H	δ^18^O	δ^2^H	δ^18^O	δ^2^H
`ST. FRANCIS RIVER AT OUTLET OF GLASIER LAKE'	`01AD003′	47.21	−68.96	21	−12.25	−86.15	−11.55	−80.90	−12.82	−90.26	−12.50	−87.43
`SAINT JOHN RIVER AT EDMUNDSTON(Flow D stop 1979)'	`01AD004′	47.36	−68.32	23	−11.30	−79.52	−10.03	−71.45	−13.37	−93.30	n.d.	n.d.
`SAINT JOHN RIVER AT GRAND FALLS'	`01AF002′	47.04	−67.74	26	−11.10	−77.95	−9.76	−67.07	−12.65	−87.70	−11.63	−81.07
`AROOSTOOK RIVER NEAR TINKER'	`01AG003′	46.82	−67.75	22	−10.44	−73.23	−8.93	−65.54	−12.01	−83.50	−10.86	−75.72
`TOBIQUE RIVER AT RILEY BROOK'	`01AH002′	47.17	−67.21	23	−11.68	−81.20	−10.68	−73.21	−13.92	−97.51	−12.38	−85.95
`MEDUXNEKEAG RIVER NEAR BELLEVILLE'	`01AJ003′	46.22	−67.73	25	−10.29	−70.66	−8.61	−57.74	−11.84	−79.93	−11.00	−74.02
`NASHWAAK RIVER AT DURHAM BRIDGE'	`01AL002′	46.13	−66.61	18	−10.43	−69.93	−9.15	−57.94	−12.30	−83.12	−11.10	−74.20
`MAGAGUADAVIC RIVER AT ELMCROFT'	`01AQ002′	45.27	−66.81	2	−9.05	−61.67	−8.39	−58.13	−9.71	−65.21	n.d.	n.d.
`RESTIGOUCHE RIVER BELOW KEDGWICK RIVER'	`01BC001′	47.67	−67.48	24	−11.91	−82.18	−10.87	−75.18	−12.54	−86.52	−12.02	−82.66
`RESTIGOUCHE RIVER ABOVE RAFTING GROUND BROOK'	`01BJ007′	47.91	−66.95	26	−12.21	−84.97	−11.30	−78.24	−13.28	−92.71	−12.57	−87.08
`SOUTHWEST MIRAMICHI RIVER AT BLACKVILLE'	`01BO001′	46.74	−65.83	25	−10.49	−71.27	−9.17	−61.23	−13.37	−91.15	−11.67	−79.14
`LITTLE SOUTHWEST MIRAMICHI RIVER AT LYTTLETON'	`01BP001′	46.94	−65.91	17	−11.15	−76.70	−9.59	−66.59	−14.67	−99.62	−12.47	−86.24

Note: n.d. = not determined; Mean=arithmetic mean, Max.=Maximum δ values, Min.=minimum δ value, Flow-weighted = weighted by discharge.

**Table 8:** Summary of isotope monitoring data for Nova Scotia.

					Mean		Max.		Min.		Flow weighted	
Station Name	Station ID	Latitude	Longitude	Samples	δ^18^O	δ^2^H	δ^18^O	δ^2^H	δ^18^O	δ^2^H	δ^18^O	δ^2^H
`CORNWALLIS RIVER AT CAMBRIDGE STATION'	`01DD002′	45.06	−64.64	1	−11.16	−73.87	−11.16	−73.87	−11.16	−73.87	n.d.	n.d.
`ST ANDREWS RIVER AT STEWIACKE'	`01DG043′	45.12	−63.35	1	−9.62	−60.58	−9.62	−60.58	−9.62	−60.58	n.d.	n.d.
`SHELBURNE RIVER AT POLLARDS FALLS BRIDGE'	`01ED013′	44.22	−65.24	1	−7.67	−46.02	−7.67	−46.02	−7.67	−46.02	n.d.	n.d.
`CHETICAMP RIVER BELOW CHETICAMP LAKE'	`01FC001′	46.65	−60.66	1	−12.15	−95.87	−12.15	−95.87	−12.15	−95.87	n.d.	n.d.
`TUSKET RIVER AT TUSKET FALLS'	`01EA001′	43.88	−65.97	2	−8.09	−49.42	−7.81	−46.57	−8.38	−52.27	n.d.	n.d.
`MERSEY RIVER BELOW GEORGE LAKE'	`01ED005′	44.33	−65.21	4	−7.69	−50.14	−6.37	−44.79	−8.67	−54.86	n.d.	n.d.
`TUSKET RIVER AT WILSONS BRIDGE'	`01EA003′	43.92	−65.87	6	−7.28	−46.33	−4.36	−31.28	−8.77	−54.56	n.d.	n.d.
`LAHAVE RIVER AT WEST NORTHFIELD'	`01EF001′	44.45	−64.59	7	−8.61	−55.23	−7.28	−47.43	−10.52	−68.20	n.d.	n.d.
`ROSEWAY RIVER AT LOWER OHIO'	`01EC001′	43.84	−65.37	8	−7.38	−47.10	−4.31	−30.84	−8.81	−56.14	n.d.	n.d.
`ST. MARYS RIVER AT STILLWATER'	`01EO001′	45.17	−61.98	8	−8.54	−54.40	−6.35	−42.22	−9.60	−60.75	n.d.	n.d.
`ANNAPOLIS RIVER AT WILMOT'	`01DC005′	44.95	−65.03	9	−9.33	−61.02	−7.77	−52.14	−10.93	−69.84	n.d.	n.d.

Note: n.d. = not determined; Mean=arithmetic mean, Max.=Maximum δ values, Min.=minimum δ value, Flow-weighted = weighted by discharge.

**Table 9:** Summary of isotope monitoring data for Prince Edward Island.

					Mean		Max.		Min.		Flow weighted	
Station Name	Station ID	Latitude	Longitude	Samples	δ^18^O	δ^2^H	δ^18^O	δ^2^H	δ^18^O	δ^2^H	δ^18^O	δ^2^H
`WEST RIVER AT RIVERDALE'	`01CC005′	46.23	−63.35	2	−10.46	−68.79	−10.40	−67.52	−10.53	−70.07	n.d.	n.d.
`DUNK RIVER AT WALL ROAD'	`01CB002′	46.35	−63.63	3	−10.46	−69.15	−10.37	−68.25	−10.51	−70.27	n.d.	n.d.

Note: n.d. = not determined; Mean=arithmetic mean, Max.=Maximum δ values, Min.=minimum δ value, Flow-weighted = weighted by discharge.

**Table 10:** Summary of isotope monitoring data for Newfoundland.

					Mean		Max.		Min.		Flow weighted	
Station Name	Station ID	Latitude	Longitude	Samples	δ^18^O	δ^2^H	δ^18^O	δ^2^H	δ^18^O	δ^2^H	δ^18^O	δ^2^H
`LITTLE MECATINA RIVER ABOVE LAC FOURMONT'	`02XA003′	52.23	−61.32	9	−15.24	−112.07	−13.45	−100.12	−17.64	−128.33	n.d.	n.d.
`UPPER HUMBER RIVER NEAR REIDVILLE'	`02YL001′	49.24	−57.36	7	−10.96	−74.58	−8.97	−60.85	−13.84	−92.18	n.d.	n.d.
`HUMBER RIVER AT HUMBER VILLAGE BRIDGE'	`02YL003′	48.98	−57.76	8	−10.42	−71.70	−10.04	−68.85	−11.35	−79.32	n.d.	n.d.
`GANDER RIVER AT BIG CHUTE'	`02YQ001′	49.02	−54.85	18	−9.31	−64.38	−8.74	−61.96	−9.78	−67.68	−9.36	−64.75
`SALMON RIVER NEAR GLENWOOD'	`02YQ005′	49.01	−54.92	2	−9.42	−65.27	−9.08	−63.88	−9.76	−66.67	n.d.	n.d.
`TERRA NOVA RIVER AT GLOVERTOWN'	`02YS005′	48.66	−54.02	25	−8.35	−58.24	−6.82	−49.53	−9.79	−66.79	−8.54	−59.34
`UGJOKTOK RIVER BELOW HARP LAKE'	`03NF001′	55.23	−61.30	10	−15.67	−116.89	−14.72	−111.33	−17.11	−126.47	−16.03	−119.79
`ATIKONAK RIVER ABOVE PANCHIA LAKE'	`03OC003′	52.97	−64.66	12	−14.65	−110.43	−13.87	−104.20	−15.59	−118.49	−14.87	−112.26
`EAST METCHIN RIVER'	`03OD007′	53.43	−63.23	6	−14.46	−107.87	−13.24	−98.71	−16.55	−123.05	n.d.	n.d.
`CHURCHILL RIVER ABOVE UPPER MUSKRAT FALLS'	`03OE001′	53.25	−60.79	9	−14.98	−112.47	−14.30	−107.07	−15.92	−118.75	n.d.	n.d.
`MINIPI RIVER BELOW MINIPI LAKE'	`03OE003′	52.61	−61.19	6	−13.59	−100.82	−12.99	−96.53	−15.05	−111.88	n.d.	n.d.
`BIG POND BROOK BELOW BIG POND'	`03OE010′	53.51	−60.29	3	−15.46	−113.43	−14.42	−106.37	−16.86	−122.07	n.d.	n.d.
`PINUS RIVER'	`03OE011′	53.15	−61.56	5	−14.96	−112.05	−12.05	−93.85	−16.84	−123.20	n.d.	n.d.
`NASKAUPI RIVER BELOW NASKAUPI LAKE'	`03PB002′	54.13	−61.43	7	−14.77	−111.86	−13.81	−106.68	−16.57	−123.10	n.d.	n.d.
`EAGLE RIVER ABOVE FALLS'	`03QC001′	53.53	−57.49	8	−14.09	−103.38	−12.46	−92.40	−16.33	−119.39	n.d.	n.d.
`ALEXIS RIVER NEAR PORT HOPE SIMPSON'	`03QC002′	52.65	−56.87	12	−13.54	−97.55	−11.71	−84.01	−16.34	−114.37	−13.92	−100.64

Note: n.d. = not determined; Mean=arithmetic mean, Max.=Maximum δ values, Min.=minimum δ value, Flow-weighted = weighted by discharge.

**Table 11:** Summary of isotope monitoring data for the Yukon Territory.

					Mean		Max.		Min.		Flow weighted	
Station Name	Station ID	Latitude	Longitude	Samples	δ^18^O	δ^2^H	δ^18^O	δ^2^H	δ^18^O	δ^2^H	δ^18^O	δ^2^H
`ALSEK RIVER ABOVE BATES RIVER'	`08AB001′	60.12	−137.98	22	−21.48	−164.73	−20.72	−160.33	−23.87	−177.88	−21.79	−167.27
`WHEATON RIVER NEAR CARCROSS'	`09AA012′	60.13	−134.88	27	−21.38	−165.17	−20.56	−159.11	−22.10	−170.78	−21.42	−165.13
`PELLY RIVER AT PELLY CROSSING'	`09BC001′	62.83	−136.58	29	−21.24	−166.95	−20.56	−162.64	−22.71	−178.15	−21.27	−166.93
`YUKON RIVER ABOVE WHITE RIVER'	`09CD001′	63.08	−139.50	28	−20.33	−159.34	−19.59	−153.65	−21.55	−170.98	−20.36	−159.51
`STEWART RIVER AT THE MOUTH'	`09DD003′	63.28	−139.25	28	−21.67	−169.66	−20.97	−164.66	−22.75	−178.42	−21.71	−169.60
`MCQUESTEN RIVER NEAR THE MOUTH'	`09DD004′	63.61	−137.27	1	−21.84	−171.20	−21.84	−171.20	−21.84	−171.20	n.d.	n.d.
`PORCUPINE RIVER AT OLD CROW'	`09FD001′	67.56	−139.88	4	−20.93	−164.20	−20.45	−161.56	−21.42	−166.57	n.d.	n.d.
`PORCUPINE RIVER NEAR INTERNATIONAL BOUNDARY'	`09FD002′	67.42	−140.89	18	−21.57	−167.95	−20.12	−156.98	−24.28	−186.17	−22.69	−175.70
`LIARD RIVER AT UPPER CROSSING'	`10AA001′	60.05	−128.91	40	−21.51	−169.55	−20.70	−164.33	−22.31	−175.66	−21.52	−169.45
`PEEL RIVER ABOVE CANYON CREEK'	`10MA001′	65.89	−136.04	21	−22.22	−172.93	−21.37	−166.83	−23.73	−183.35	−22.76	−176.35

Note: n.d. = not determined; Mean=arithmetic mean, Max.=Maximum δ values, Min.=minimum δ value, Flow-weighted = weighted by discharge.

**Table 12:** Summary of Isotope monitoring data for the Northwest Territories.

					Mean		Max.		Min.		Flow weighted	
Station Name	Station ID	Latitude	Longitude	Samples	δ^18^O	δ^2^H	δ^18^O	δ^2^H	δ^18^O	δ^2^H	δ^18^O	δ^2^H
`HAY RIVER NEAR HAY RIVER'	`07OB001′	60.74	−115.86	20	−17.53	−145.53	−14.62	−131.24	−19.51	−157.03	−17.90	−147.24
`LOCKHART RIVER AT OUTLET OF ARTILLERY LAKE'	`07RD001′	62.89	−108.47	10	−17.19	−142.46	−13.81	−127.90	−17.84	−146.14	−17.58	−144.06
`SNARE RIVER BELOW GHOST RIVER'	`07SA002′	63.97	−115.43	17	−16.90	−144.96	−15.92	−138.55	−24.51	−200.20	−16.41	−141.26
`YELLOWKNIFE RIVER AT OUTLET OF PROSPEROUS LAKE'	`07SB002′	62.56	−114.22	4	−14.51	−130.61	−14.30	−128.85	−14.65	−131.54	n.d.	n.d.
`YELLOWKNIFE RIVER AT INLET TO PROSPEROUS LAKE'	`07SB003′	62.67	−114.26	9	−14.68	−132.33	−14.09	−129.68	−15.03	−134.17	n.d.	n.d.
`BAKER CREEK AT OUTLET OF LOWER MARTIN LAKE'	`07SB013′	62.51	−114.41	7	−13.93	−131.42	−9.73	−108.56	−24.89	−198.24	n.d.	n.d.
`LA MARTRE RIVER BELOW OUTLET OF LAC LA MARTRE'	`07TA001′	63.11	−116.97	16	−15.11	−133.88	−14.07	−128.15	−16.89	−144.18	−15.02	−133.36
`FLAT RIVER NEAR THE MOUTH'	`10EA003′	61.53	−125.41	5	−22.21	−172.79	−21.76	−169.63	−22.85	−176.81	n.d.	n.d.
`SOUTH NAHANNI RIVER ABOVE VIRGINIA FALLS'	`10EB001′	61.64	−125.80	24	−22.47	−174.37	−21.51	−165.86	−23.22	−179.04	−22.25	−172.63
`PRAIRIE CREEK AT CADILLAC MINE'	`10EC002′	61.56	−124.81	1	−22.53	−173.27	−22.53	−173.27	−22.53	−173.27	n.d.	n.d.
`LIARD RIVER AT FORT LIARD'	`10ED001′	60.24	−123.48	27	−21.22	−165.70	−20.00	−157.00	−23.37	−181.52	−21.84	−169.95
`LIARD RIVER NEAR THE MOUTH'	`10ED002′	61.74	−121.23	32	−21.08	−164.61	−17.26	−140.89	−23.40	−180.28	−21.30	−165.91
`BIRCH RIVER AT HIGHWAY NO. 7'	`10ED003′	61.33	−122.09	20	−20.45	−161.98	−18.57	−150.52	−23.80	−181.99	−20.86	−165.40
`BLACKSTONE RIVER AT HIGHWAY NO. 7′	`10ED007′	61.06	−122.90	9	−20.92	−164.44	−19.59	−155.15	−23.69	−185.46	n.d.	n.d.
`SCOTTY CREEK AT HIGHWAY NO. 7'	`10ED009′	61.42	−121.46	24	−20.05	−159.95	−18.01	−147.42	−23.66	−184.72	−21.64	−169.99
`TROUT RIVER AT HIGHWAY NO. 1′	`10FA002′	61.14	−119.84	16	−18.46	−151.45	−17.44	−145.01	−20.41	−161.77	−17.80	−147.40
`JEAN-MARIE RIVER AT HIGHWAY NO. 1'	`10FB005′	61.45	−121.24	18	−20.11	−161.07	−19.06	−153.27	−22.28	−173.97	−21.61	−170.10
`MACKENZIE RIVER AT STRONG POINT'	`10FB006′	61.82	−120.79	16	−17.29	−141.24	−16.88	−138.52	−19.63	−158.32	−17.49	−142.53
`ROOT RIVER NEAR THE MOUTH'	`10GA001′	62.48	−123.43	22	−22.43	−174.64	−21.14	−165.68	−26.21	−202.73	−23.41	−182.02
`WILLOWLAKE RIVER BELOW METAHDALI CREEK'	`10GB001′	62.65	−122.91	1	−22.34	−173.60	−22.34	−173.60	−22.34	−173.60	n.d.	n.d.
`WILLOWLAKE RIVER ABOVE METAHDALI CREEK'	`10GB006′	62.65	−122.90	23	−18.30	−151.61	−16.10	−138.64	−24.02	−188.21	−21.71	−171.17
`MACKENZIE RIVER AT FORT SIMPSON'	`10GC001′	61.87	−121.36	7	−17.41	−141.30	−16.73	−138.62	−18.89	−147.79	n.d.	n.d.
`MARTIN RIVER AT HIGHWAY NO. 1′	`10GC003′	61.89	−121.61	2	−21.58	−171.97	−19.44	−159.18	−23.71	−184.76	n.d.	n.d.
`KEELE RIVER ABOVE TWITYA RIVER'	`10HA004′	64.14	−128.21	22	−22.88	−177.87	−21.97	−172.48	−24.46	−191.20	−22.91	−177.60
`REDSTONE RIVER 63 KM ABOVE THE MOUTH'	`10HB005′	63.92	−125.30	13	−22.83	−177.67	−21.64	−169.54	−25.35	−198.70	−22.89	−177.87
`CAMSELL RIVER AT OUTLET OF CLUT LAKE'	`10JA002′	65.60	−117.76	5	−16.76	−144.35	−16.44	−142.62	−17.19	−146.24	n.d.	n.d.
`GREAT BEAR RIVER AT OUTLET OF GREAT BEAR LAKE'	`10JC003′	65.13	−123.55	22	−18.44	−153.34	−18.16	−151.27	−18.81	−155.83	−18.44	−153.14
`GREAT BEAR LAKE AT HORNBY BAY'	`10JE002′	66.60	−117.62	1	−17.18	−146.45	−17.18	−146.45	−17.18	−146.45	n.d.	n.d.
`MACKENZIE RIVER AT NORMAN WELLS'	`10KA001′	65.27	−126.85	36	−18.97	−153.72	−18.17	−149.05	−21.18	−168.19	−19.32	−155.43
`ARCTIC RED RIVER NEAR THE MOUTH'	`10LA002′	66.79	−133.09	2	−21.46	−168.19	−21.29	−167.04	−21.63	−169.33	n.d.	n.d.
`MACKENZIE RIVER AT ARCTIC RED RIVER'	`10LC014′	67.46	−133.75	19	−18.79	−151.25	−18.06	−146.31	−20.50	−162.28	−19.15	−153.57
`PEEL RIVER ABOVE FORT MCPHERSON'	`10MC002′	67.26	−134.89	18	−21.79	−169.75	−19.38	−155.37	−23.16	−179.85	−21.73	−169.20
`ANDERSON RIVER BELOW CARNWATH RIVER'	`10NC001′	68.63	−128.42	2	−17.63	−148.24	−16.99	−146.11	−18.26	−150.37	n.d.	n.d.
`COPPERMINE RIVER BELOW DESTEFFANY LAKE'	`10PA001′	64.62	−111.95	10	−18.11	−148.69	−17.40	−144.62	−18.91	−152.35	n.d.	n.d.
`COPPERMINE RIVER AT OUTLET OF POINT LAKE'	`10PB001′	65.42	−114.01	1	−18.60	−150.47	−18.60	−150.47	−18.60	−150.47	n.d.	n.d.
`POINT LAKE NEAR THE OUTLET'	`10PB003′	65.40	−113.99	9	−18.44	−150.57	−17.63	−144.89	−19.31	−156.72	n.d.	n.d.

Note: n.d. = not determined; Mean=arithmetic mean, Max.=Maximum δ values, Min.=minimum δ value, Flow-weighted = weighted by discharge.

**Table 13:** Summary of isotope monitoring data for Nunavut.

					Mean		Max.		Min.		Flow weighted	
Station Name	Station ID	Latitude	Longitude	Samples	δ^18^O	δ^2^H	δ^18^O	δ^2^H	δ^18^O	δ^2^H	δ^18^O	δ^2^H
`THLEWIAZA RIVER ABOVE OUTLET SEALHOLE LAKE'	`06HB002′	60.78653	−98.7769	5	−15.5517	−127.23	−15.4288	−126.18	−15.7028	−128.278	n.d.	n.d.
`THELON RIVER ABOVE BEVERLY LAKE'	`06JC002′	64.53039	−101.362	10	−17.8962	−144.819	−16.5847	−137.216	−19.567	−155.816	n.d.	n.d.
`DUBAWNT RIVER AT OUTLET OF MARJORIE LAKE'	`06KC003′	64.23242	−99.4761	7	−16.9955	−138.282	−16.7233	−136.116	−17.2771	−140.063	n.d.	n.d.
`KAZAN RIVER AT OUTLET OF ENNADAI LAKE'	`06LA001′	61.25361	−100.974	3	−15.8945	−131.017	−15.7362	−129.783	−16.1915	−133.368	n.d.	n.d.
`KAZAN RIVER ABOVE KAZAN FALLS'	`06LC001′	63.65258	−95.8521	8	−17.5774	−140.589	−16.727	−134.053	−19.4905	−152.791	n.d.	n.d.
`THELON RIVER BELOW OUTLET OF SCHULTZ LAKE'	`06MA006′	64.77828	−97.0539	8	−18.2358	−146.321	−17.6855	−142.155	−18.5955	−149.104	n.d.	n.d.
`IZOK LAKE INFLOW'	`10PB002′	65.63972	−112.863	1	−18.4293	−150.323	−18.4293	−150.323	−18.4293	−150.323	n.d.	n.d.
`COPPERMINE RIVER ABOVE COPPER CREEK'	`10PC004′	67.22833	−115.889	3	−20.3613	−162.922	−18.8468	−153.677	−21.409	−170.237	n.d.	n.d.
`BURNSIDE RIVER NEAR THE MOUTH'	`10QC001′	66.72625	−108.813	7	−19.9399	−159.031	−19.1189	−155.052	−20.9862	−166.263	n.d.	n.d.
`ELLICE RIVER NEAR THE MOUTH'	`10QD001′	67.70833	−104.139	7	−19.9016	−158.244	−18.3219	−147.682	−21.5546	−169.835	n.d.	n.d.
`BACK RIVER BELOW BEECHY LAKE'	`10RA001′	65.18722	−106.086	15	−19.3473	−155.762	−18.4722	−149.695	−21.3573	−169.593	−20.1158	−160.195
`BAILLIE RIVER NEAR THE MOUTH'	`10RA002′	65.01056	−104.491	14	−19.2326	−154.954	−17.4058	−143.806	−21.3923	−167.922	−20.7189	−163.12
`BACK RIVER ABOVE HERMANN RIVER'	`10RC001′	66.08614	−96.5108	9	−19.5286	−154.728	−18.9855	−150.704	−20.0274	−158.325	n.d.	n.d.

Note: n.d. = not determined; Mean=arithmetic mean, Max.=Maximum δ values, Min.=minimum δ value, Flow-weighted = weighted by discharge.

## 2 Experimental Design, Materials and Methods

### 2.1 Network design

331 stations were selected in cooperation with WSC to establish full national coverage and to incorporate a range of watershed scales for various stakeholder applications. The temporal frequency of sampling ranged from 1 to 12 times per year, with schedules constrained by the frequency of visits by technical staff. A core network of 161 stations had more than 12 samples collected for isotopic analysis during 2013–2019 and had available discharge data to enable meaningful flow weighting of the isotopic signatures. Overall, the network included watersheds ranging in size from 100 to greater than 10,000 km^2^ and situated across 90° longitude and 25° latitude.

### 2.2 Field methods and sampling

Water samples were collected from mid-channel locations at mid-depth within the river water column at each gauging station to ensure representativeness of discharge. If this was not possible due to ice conditions or other safety issues, staff typically collected samples from an adjacent bank, avoiding poorly mixed zones below tributaries and/or backwater areas. Alternately, water was collected during ice-on conditions from a borehole augered in the ice. Metadata such as station number, sampling date, time, backwater effects, and ice conditions were routinely recorded. Water samples were collected in 30 mL high density polyethylene (HDPE) bottles with tightly sealed lids to prevent evaporation, and stored at room temperature prior to and during shipment to the lab in Victoria. Freezing of samples was avoided. Sample bottles were labelled in the field with station number, date, time, ice conditions, and name of sampler, as well as any applicable comments. A list of samples was also generally provided to InnoTech Alberta by field staff.

Locations for sampling were selected in consultation with the Water Survey staff from the network of active monitoring stations, based on considerations such as coverage, basin size, and anticipated applications for regional stakeholders such as Indigenous groups, hydroelectric generating plants, oil sands and mining operators, recreation, and municipal, provincial and national governments. In general, water samples were collected either as mid-depth, mid-channel grab samples, or dip samples from shore, bridges or cableways. In the case of poor mixing conditions or difficult access, sites were reviewed and a specific plan was developed with water technicians familiar with each site. Locations were selected to maximize the potential for obtaining a representative sample based on knowledge of the mixing conditions of the river at each station, and their seasonal variability including differences during ice-on, melt periods, and ice-break-up conditions. Locations downstream from major tributaries were avoided where possible due to potential for incomplete lateral mixing. By coordinating water isotope sampling with WSC gauge locations where discharge is measured, we sought to develop a dataset where both discharge and isotopic composition was known to allow for flow weighting of the isotopic signatures. This is often the preferred method for obtaining long-term isotope values for continental runoff, although we evaluate the significance of this approach later by comparing flow-weighting and simple averaging.

### 2.3 Laboratory analysis

All isotope results were analyzed by isotope ratio mass spectrometry using a Thermo Scientific Delta V Advantage located at InnoTech, Victoria. Oxygen was prepared using a Gasbench II by equilibrating water and CO_2_ and then introducing CO_2_ into the dual inlet using an autosampler [21]. Hydrogen was analyzed by auto-injecting water into a chromium reactor heated to 875°C in the HDevice to produce H_2_, which was streamed to the dual inlet for analysis [22]. In all cases, analyses were made within one year of sample collection as confirmed to be appropriate for HDPE bottles [23]. Results are reported in “δ” notation in permil (‰) relative to Vienna Standard Mean Ocean Water (V-SMOW) and normalized to the SMOW-SLAP scale where SLAP is Standard Light Arctic Precipitation [24]. Analytical uncertainty estimated based on 2-σ of repeats is ±0.14 for δ^18^O (*n* = 382) and ±0.52 for δ^2^H (*n* = 450).

## Ethics Statement

We hereby assert that the manuscript is the authors' own original work, which has not been previously published elsewhere, nor is it currently being considered elsewhere for publication. The paper reflects the authors' own research and analysis in a truthful and complete manner, and properly credits the meaningful contributions of co-authors and co-researchers. We have sought to appropriately place the results in the context of prior and existing research and have properly disclosed all sources. All authors have contributed to the work and will take public responsibility for its content.

## Credit Author Statement

**J.J. Gibson:** Conceptualization, Data Curation, Methodology, Formal Analysis, Validation, Writing, Editing, Project Administration, Funding Acquisition; **P. Eby:** Laboratory Analysis; **T.A. Stadnyk:** Conceptualization, Writing, Editing, Review; **T. Holmes:** Spatial Analysis, Coding, Visualization, Formal Analysis, Review; **S.J. Birks:** Conceptualization, Review; **A. Pietroniro:** Conceptualization, Project Administration, Field Coordination, Funding Acquisition, Review.

## Declaration of Competing Interest

The authors declare that they have no known competing financial interests or personal relationships which have or could be perceived to have influenced the work reported in this article.

## Appendix Supplementary materials

Image, application 1

## Footnotes

Supplementary material associated with this article can be found in the online version at doi:10.1016/j.dib.2021.106723.
